# Ubiquitin-dependent regulation of Cdc42 by XIAP

**DOI:** 10.1038/cddis.2017.305

**Published:** 2017-06-29

**Authors:** Arun Murali, Jaeyoung Shin, Hajime Yurugi, Aswini Krishnan, Masato Akutsu, Alejandro Carpy, Boris Macek, Krishnaraj Rajalingam

**Affiliations:** 1 Molecular Signaling Unit-FZI, Institute of immunology, University Medical Center Mainz, JGU-Mainz, Germany; 2 BMLS, Goethe University, Frankfurt, Germany; 3 Proteome Center Tuebingen, Interfaculty Institute for Cell Biology, University of Tuebingen, Tuebingen, Germany

## Abstract

Rho GTPases control fundamental cellular processes and Cdc42 is a well-studied member of the family that controls filopodia formation and cell migration. Although the regulation of Cdc42 activity by nucleotide binding is well documented, the mechanisms driving its proteostasis are not clear. Here, we demonstrate that the highly conserved, RING domain containing E3 ubiquitin ligase XIAP controls the protein stability of Cdc42. XIAP binds to Cdc42 and directly conjugates poly ubiquitin chains to the Lysine 166 of Cdc42 targeting it for proteasomal degradation. Depletion of XIAP led to an increased protein stability and activity of Cdc42 in normal and tumor cells. Consistently, loss of XIAP enhances filopodia formation in a Cdc42-dependent manner and this phenomenon phenocopies EGF stimulation. Further, XIAP depletion promotes lung colonization of tumor cells in mice in a Cdc42-dependent manner. These observations shed molecular insights into ubiquitin-dependent regulation of Cdc42 and that of actin cytoskeleton.

Rho GTPases are evolutionarily conserved proteins and make up a unique subfamily within the Ras superfamily of GTPases. They are characterized by the presence of a ‘Rho insert domain’, which is located between the fifth *β*-strand and the fourth *α*-helix of the Rho GTPase.^1^ Like other GTPases, most members of the Rho GTPase family cycle between an inactive GDP bound and an active GTP-bound form. This conversion is catalyzed by guanine nucleotide exchange factors (GEFs), which release bound GDP and facilitates the binding of GTP, and GTPase-activating proteins (GAPs), which inactivate the GTPases by hydrolyzing the GTP back to GDP. Yet another mechanism of inactivating the Rho GTPases is by chaperones called Rho guanine nucleotide dissociation inhibitors (RhoGDIs) that bind to Rho GTPases in the cytosol and sequester them from binding to effectors as well as prevent them from proteolytic degradation.^2^ Rho GTPases can also be regulated by ubiquitination and degradation.^3, 4, 5, 6^

Cdc42 is one of the better-studied members of the Rho GTPase family along with Rac1 and RhoA. It has been implicated in a variety of cellular processes including cell motility, cytoskeletal reorganization, filopodia formation, polarity and cell cycle progression. Although the role of Cdc42 in promoting filopodia seems to be cell type specific, several lines of evidence suggest a crucial role for this Rho GTPase in filopodia formation.^7, 8^ Further, epidermal growth factor (EGF) has been implicated in Cdc42-dependent protrusion formation.^9^ Here we demonstrate that an evolutionarily conserved member of the inhibitor of apoptosis protein (IAP) family, XIAP functions as a direct E3 ubiquitin ligase of Cdc42 thus controlling its protein stability.

IAPs are a family of multi-functional proteins, primarily characterized by the presence of a baculoviral IAP repeat (BIR) domain, which are classical protein–protein interaction motifs.^10^ Among the eight mammalian IAPs known (X-linked inhibitor of apoptosis protein (XIAP), cellular IAP1/2 (cIAP1 and c-IAP2), melanoma inhibitor of IAP (ML-IAP), Survivin, NAIP, BRUCE and ILP2), five of them also contain a RING finger carboxy terminal domain that confers them with E3 ubiquitin ligase activity.^11^ Ubiquitylation is the most versatile form of post-translational modification wherein Ubiquitin (Ub), a 76 amino acid protein is covalently conjugated to target proteins in a three-step process involving E1, E2 and E3 enzymes.^12, 13^ Conjugation of ubiquitin either as a single moiety or as chains of different kinds results in profound effects in cells ranging from protein stability, localization of target proteins to formation of dynamic signaling complexes thus controlling virtually every cellular process.^14^

Apart from the traditional role of IAPs in regulating cell death, recent studies revealed a crucial role of IAPs in controlling inflammation, migration and differentiation.^15^ IAPs (especially XIAP, cIAP1/2 and ML-IAP) were discovered to negatively regulate the stability of CRAF kinase and cell migration.^16^ Further, our recent work has shown that XIAP and cIAP1 can control the plasticity of cell migration by directly regulating the ubiquitination and proteasomal degradation of the Rho GTPase Rac1.^4^ In addition, XIAP can directly conjugate K-63 linked ubiquitin chains to MEKK2 and MEKK3 and interfere with the ERK5 signaling cascade and human myogenic differentiation.^17^ Although the control of Cdc42 activity by GEFs and GAPs are known and well studied, the molecular mechanisms underpinning the protein degradation of Cdc42 are not clear to date. Here we show that XIAP contributes to Cdc42 homeostasis by direct ubiquitination leading to protein degradation thus controlling filopodia dynamics and tumor cell metastases. These studies further unveil yet another facet of IAP biology in controlling actin cytoskeleton and tumor metastases.

## Results

**Figure 1:**
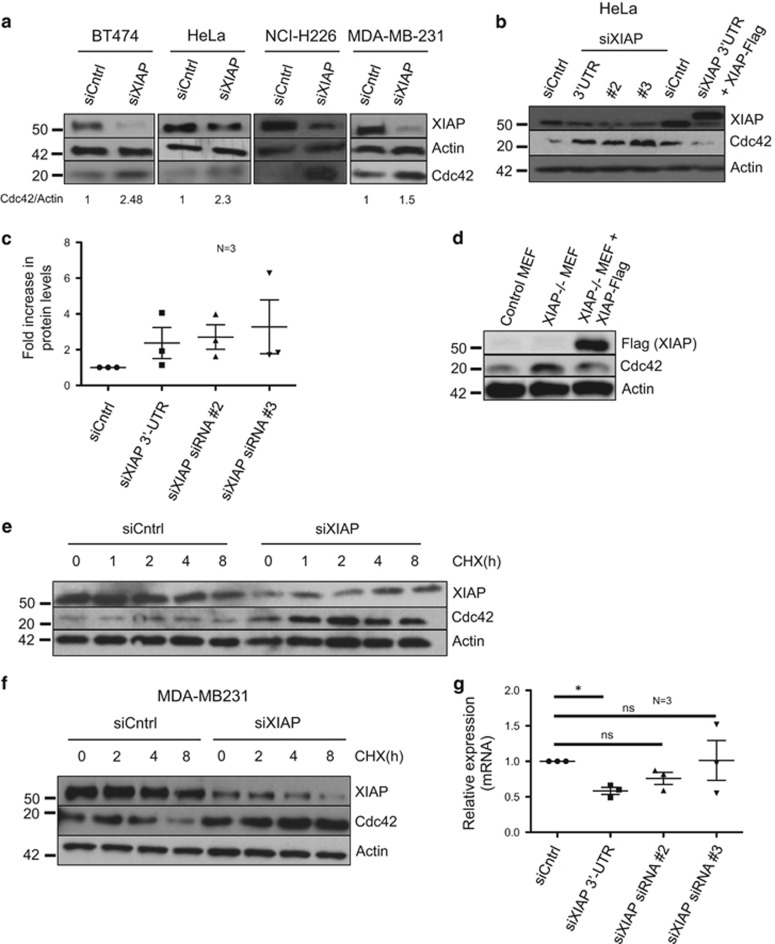
Depletion of IAPs leads to up-regulation of Cdc42 (**a**) BT474, HeLa, NCI-H226 and MDA-MB231 cells were transfected with Control or XIAP siRNA for 48 h and lysates were then collected for immunoblotting. The levels of total Cdc42 were monitored in the immunoblots. Quantification was performed by densitometry (as described in Materials and Methods). (**b**) HeLa cells were transfected with three different XIAP siRNAs and Cdc42 levels were monitored. Further, HeLa cells transfected with the 3’-UTR XIAP siRNA were complemented with the XIAP-Flag vector and Cdc42 levels were observed. (**c**) Quantification of three independent experiments where three different XIAP siRNAs were used and Cdc42 protein expression was checked 48 h post transfection (**d**) Mouse embryonic fibroblasts (MEFs) were cultured and lysed to check for Cdc42 levels. Control MEFs and XIAP knockout MEFs stably complemented with Flag tagged XIAP were employed. (**e**, **f**) Cycloheximide chases were performed in HeLa (**e**) and MDA-MB231 (**f**), and the stabilization of Cdc42 upon depletion of XIAP was observed. (**g**) qPCR performed with the three different XIAP siRNAs to observe the transcriptional regulation of Cdc42. Data shown are from three independent experiments

We have previously shown that IAPs, especially XIAP can directly control actin cytoskeleton by regulating Rac1 stability.^4^ We often detected that XIAP-depleted cells exhibited several actin-rich protrusions irrespective of the matrices on which the cells were grown. We thus hypothesized that XIAP might regulate other related Rho GTPases like Cdc42. We first checked whether depletion of XIAP led to any alterations in Cdc42 protein levels. Interestingly, depletion of XIAP led to a striking increase in Cdc42 levels in multiple cell types (Figure 1a). Depletion of XIAP with multiple siRNAs led to a similar increase in Cdc42 levels (Figures 1b and c). Further, complementation experiments revealed that expression of XIAP in trans prevented the increase in Cdc42 levels in cells transfected with a siRNA targeted against the 3’UTR of the XIAP mRNA (Figure 1b). Although we consistently detect an increase in the protein levels of Cdc42 upon XIAP knockdown with multiple siRNAs we do not observe a linear correlation between the extent of XIAP depletion and increase in Cdc42 levels. To further corroborate these observations we tested Mouse Embyronic Fibroblasts derived from XIAP-deficient mice. XIAP-deficient MEFs exhibited high Cdc42 protein level, which is reduced after complementation of the same cells with exogenously expressed XIAP cDNA (Figure 1d). Taken together, these results confirm the specificity of the observed phenotype. We performed cycloheximide chase experiments in two different cell lines, HeLa and MDA-MB231 and we detected that loss of XIAP led to increased protein stability of Cdc42 (Figures 1e and f). However, loss of XIAP did not lead to an increase in the mRNA levels of Cdc42 despite strong increase in the protein levels, suggesting that XIAP directly contributes to the proteostasis of Cdc42 (Figure 1g) in multiple cell types.

**Figure 2:**
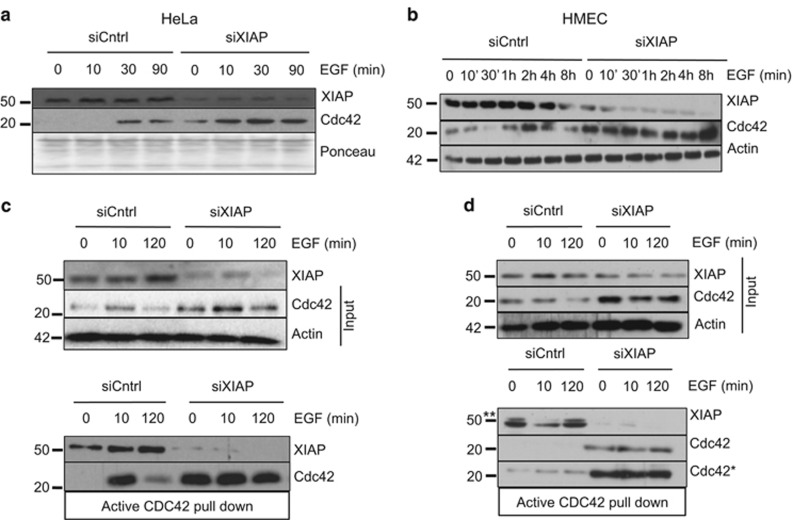
EGF treatment further enhances the stability of Cdc42 upon XIAP depletion (**a**) HeLa cells and (**b**) HMECs were transfected with XIAP siRNA and treated with EGF 48 h post transfection for the times indicated in the figure. The levels of Cdc42 were monitored via immunoblotting. (**c**, **d**) Active Cdc42 pulldown using GST PAK-PBD protein was performed in HeLa and HMECs to test for levels of the active protein upon EGF treatment at times indicated in the figure. * denotes a higher exposure of Cdc42 in (**d**). **denotes the upper non-specific band in the XIAP blot

We often detected that stimulation of either HeLa cells or primary HMEC cells with EGF kinetically led to an increase in the protein levels of Cdc42 (Figures 2a and b). Interestingly, XIAP-depleted cells exhibited high Cdc42 levels despite stimulation with EGF (Figures 2a and b). We then investigated whether XIAP depletion also affected the activation status of Cdc42 in cells. CRIB pulldown assays employing PAK-PBD in control and XIAP-depleted cells showed that the loss of XIAP also led to an increase in the GTP-bound active form of Cdc42 in normal as well as in tumor cells (Figures 2c and d). Intriguingly, we also detected XIAP co-precipitating with active Cdc42 suggesting that XIAP and Cdc42 co-exist in a protein complex (Figures 2c and d). We then tested for phenotypic changes in XIAP-depleted cells as Cdc42 has been well documented to control filopodia formation in multiple cell types.

**Figure 3:**
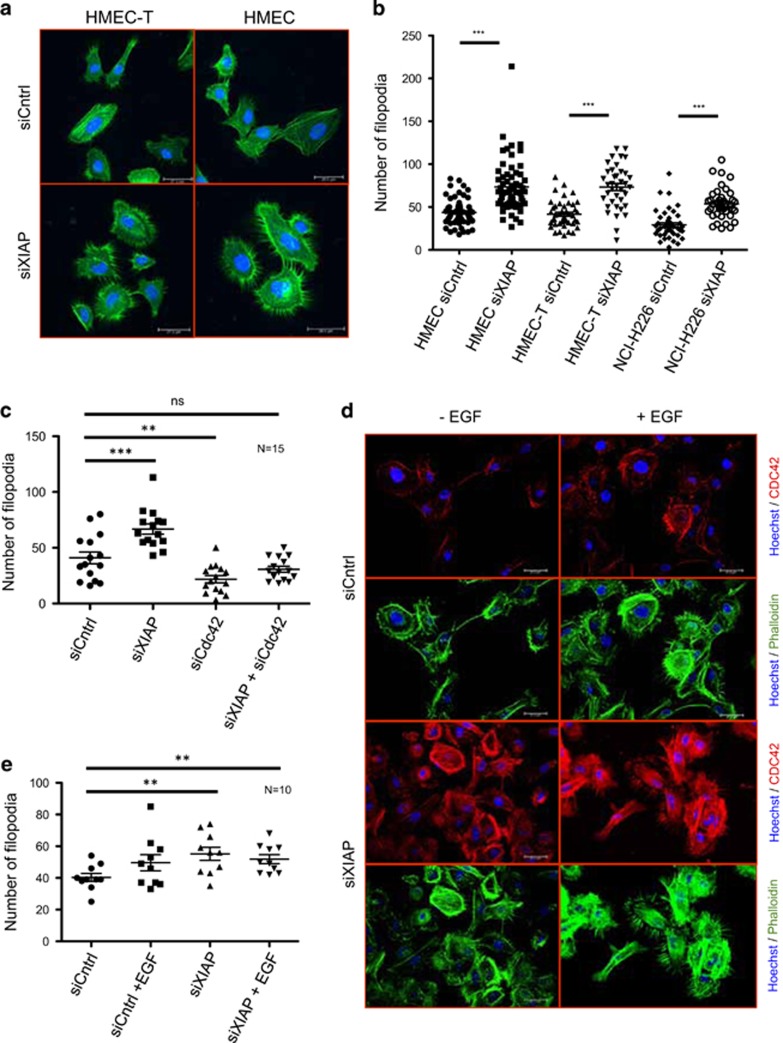
Depletion of XIAP increases the number of Filopodia (**a**) HMEC, NCI-H226 and HMEC-T cells were depleted of XIAP and immunofluorescence staining was performed using Phalloidin AlexFluor 488 to look for differences in actin structures. (**b**) Quantification of the number of filopodia in the three cell lines of (**a**) upon depletion of XIAP. Data shown are from three independent experiments with a total of 40–60 cells counted per condition (see Materials and Methods for details), *P*<0.001 (**c**) HMEC-T cells were depleted of XIAP as well as Cdc42, the quantification of the number of filopodia is shown, *n*=15 cells. (**d**) HMEC-T cells were treated with EGF stimulation after Control or XIAP siRNA treatment and immunofluorescence was performed using three different stains: Phalloidin for Actin, Hoechst for the nucleus and a Cdc42 primary antibody followed by Cy3 mouse secondary to check for localization of Cdc42 protein in the cell (**e**) Quantification of the number of filopodia seen in Figure (**d**) with *n*=10 cells counted per condition, *P*<0.01

As expected, XIAP depletion led to profuse filopodia formation in both primary and immortalized HMEC cells (Figures 3a and b). We also observed the same phenomenon in other tumor cell lines such as NCI-H226 (Figure 3b and Supplementary Figure 1A). Further, co-depletion of Cdc42 reduced the number of filopodia in both control and XIAP-depleted cells confirming the specificity of this phenotype (Figure 3c and Supplementary Figures 1B-1D). We detected that XIAP-depleted cells phenocopied EGF stimulated cells with an increased Cdc42 activity (Figures 3d and e). Immunocytochemical studies employing a validated Cdc42 antibody revealed that Cdc42 is strongly upregulated in XIAP-depleted cells and localizes to the filopodial protrusions in immortalized HMEC cells (Figure 3d). These results confirmed that loss of XIAP led to an increase in the total as well as active levels of Cdc42 thus contributing to the enhanced actin-rich protrusions in these cells.

**Figure 4:**
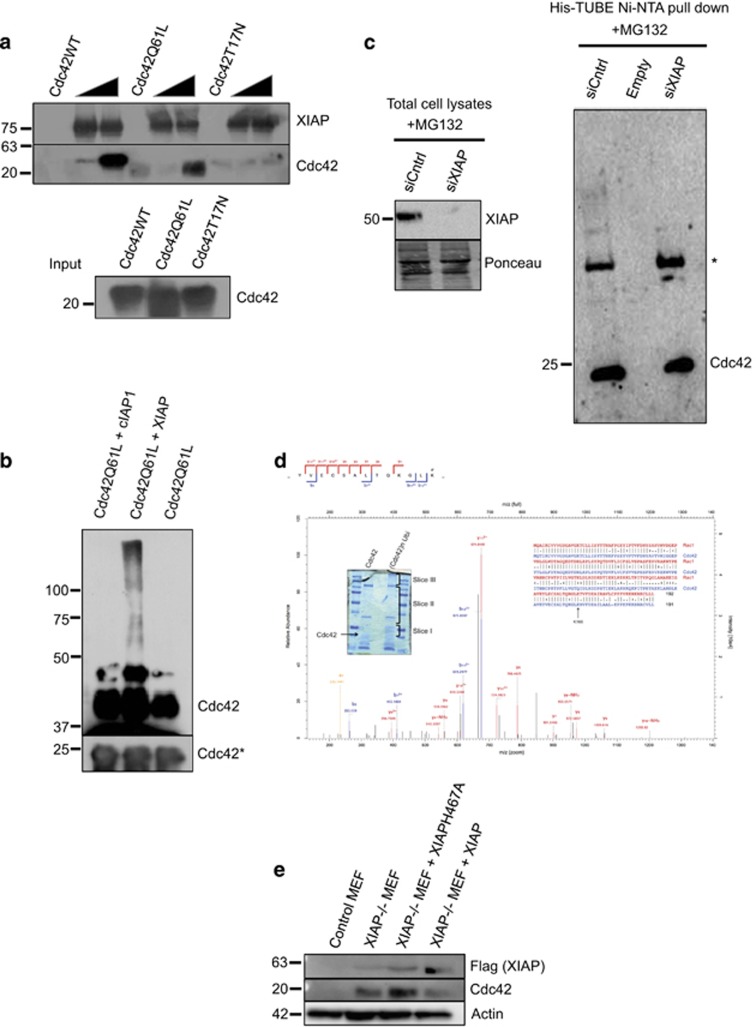
(**a**) Interaction between XIAP and different Cdc42 mutants was tested via an *in vitro* GST Pulldown assay. With GST protein as a control, two different concentrations of cleaved Cdc42 mutants were used to test the binding to GST tagged XIAP (**b**) *In vitro* ubiquitination of Cdc42 by XIAP. Purified recombinant Cdc42Q61L was subjected to *in vitro* ubiquitination by XIAP and cIAP1 recombinant proteins (protocol described in Materials and Methods). (**c**) HeLa cells were transfected with XIAP siRNA for 48 h and treated with MG132 for 6 h. The cells were lysed in RIPA buffer and His-TUBE immobilized on Ni-NTA beads were employed to enrich the ubiquitinated proteins. The samples were loaded onto a gel and the presence of Cdc42 was monitored by immunoblots. The efficiency of XIAP knockdown was tested in the lysates control. * denotes an unspecific band. (**d**) Gel slices of the *in vitro* ubiquitination reaction were subjected to mass spectrometric analysis to determine the Lysine(s) responsible for the ubiquitination. Inset shows the gel slices taken for the analysis as well as a comparison between the sequences of Rac1 and Cdc42 (**e**) Mouse embryonic fibroblasts (MEFs) were cultured and lysed to check for Cdc42 levels. Control MEFs and XIAP knockout MEFs stably complemented with different XIAP constructs were used for this experiment. * denotes lower exposure of Cdc42

We then investigated the molecular mechanisms behind XIAP-mediated Cdc42 degradation. Interestingly, we detected that XIAP directly binds to Cdc42 in a RING domain-dependent manner (Supplementary Figure 2A). XIAP failed to interact with the Cdc42T17N mutant *in vitro,*suggesting that the observed interaction may be dependent on the activation status of Cdc42 (Figure 4a). We then explored the possibility whether XIAP is a direct E3 ubiquitin ligase of Cdc42. *In vitro* ubiquitination experiments revealed that XIAP could directly conjugate ubiquitin to wild-type and CdcQ61L mutants efficiently (Supplementary Figure 2B), consistent with the interaction experiments. Further experiments also revealed that XIAP but not cIAP1 could directly conjugate poly ubiquitin chains to the active Cdc42Q61L mutant (Figure 4b). We then tested if XIAP can influence the ubiquitination and proteasomal degradation of Cdc42 at endogenous levels. To pursue these studies, we performed pulldown experiments including TUBEs (tandem ubiquitin binding entity) to enrich ubiquitinated proteins from control and XIAP-depleted cell lysates as mentioned in the methods section. Interestingly, we detected that loss of XIAP reduced the polyubiquitination of Cdc42 at endogenous levels (Figure 4c). Previous studies with bacterial toxins have suggested that activated Rho GTPases are often targeted for proteosomal degradation.^18^ We then tested if persistent activation of Cdc42 led to its decreased protein stability. Consistent with the observations made with Rac1, cycloheximide chase experiments revealed that the activated Q61L form of Cdc42 was less stable when compared to either wild-type Cdc42 or the Cdc42T17N inactive mutants (Supplementary Figure 2C).

We then employed mass spectrometric analysis to identify the possible ubiquitination sites of Cdc42. These analyses revealed that XIAP conjugated ubiquitin chains to the K166 of Cdc42 which is localized to the C-terminus of the Rho GTPase (Figure 4d). Consistently, mutation of K166 (Cdc42Q61LK166A) significantly enhanced the protein stability of Cdc42Q61L suggesting that ubiquitination at this site by XIAP is relevant for the proteolysis of activated Cdc42 (Supplementary Figure 2D).

As XIAP can directly ubiquitinate Cdc42, we tested if the RING domain of XIAP is required for modulating the protein levels of Cdc42 *in vivo*. To perform these studies, we employed MEFs derived from XIAP-deficient mice and complimented them with either wild-type or RING (XIAPH467A) mutants. Interestingly, expression of wild-type XIAP but not the RING mutant of XIAP reduced the Cdc42 levels in XIAP-deficient MEFs (Figure 4e). In fact, expression of the RING mutant of XIAP augmented the protein levels of Cdc42 under these settings (Figure 4e). These results further confirm that XIAP directly influences the protein stability of Cdc42 through its E3 ligase activity.

**Figure 5:**
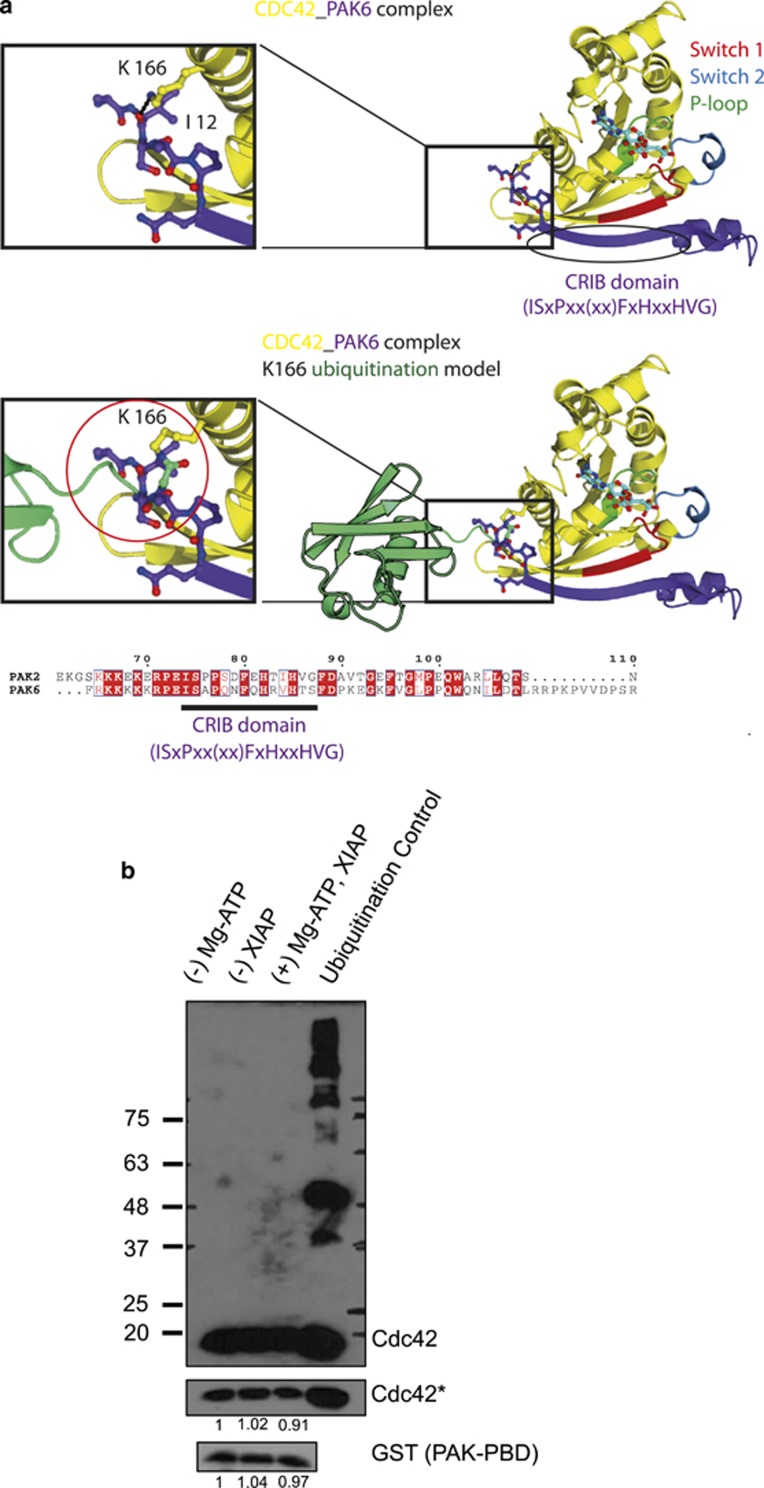
(**a**) Ribbon model of crystal structure of CDC42 (Yellow) in complex with PAK6 (Purple) (PDB: 2ODB). P-loop (residues 10-17), Switch I (residues 32–40), and switch II (residues 60–67) of Cdc42 are green, red and cyan, respectively. Nucleotide (cyan), K166 of CDC42 and residues 11-16 of PAK6 are shown in ball-and-stick representation. Hydrogen bond between K166 of CDC42 and PAK6 (carbonyl oxygen of I12) is shown as black dashed line. The second figure shows a model of ubiquitinated (Green) CDC42 (Yellow) in complex with PAK6 (Purple). Finally, a sequence alignment of CRIB domain of PAK2 and PAK6. (**b**) The same *in vitro* ubiquitination assay as in Figure 4b was performed and the reaction was then subjected to a PAK-PBD GST pulldown (as described in Materials and Methods) to check for interaction of ubiquitinated Cdc42 and its downstream effector PAK. The ubiquitination of Cdc42 was confirmed in the samples employed for PAK-PBD pulldown (lane 4)

We performed molecular modeling studies based on the published crystal structures of Cdc42 and PAK6, which led to the prediction that the conjugation of Ubiquitin to K166 could disrupt the interaction between Cdc42 and its effector protein PAK (Figure 5a). Consistently, CRIB pulldown experiments involving the p21-binding domain (PBD) of PAK revealed that ubiquitinated Cdc42 failed to interact with PAK-PBD. Cdc42 was first subjected to an *in vitro* ubiquitination assay and GST PAK-PBD was subsequently added to the reaction mix and the CRIB pulldown was performed as described in the methods. We observed that ubiquitinated Cdc42 failed to interact with PAK-PBD, suggesting that conjugation of ubiquitin to K166 directly prevented the interaction of Cdc42 with its downstream effector (Figure 5b).

**Figure 6:**
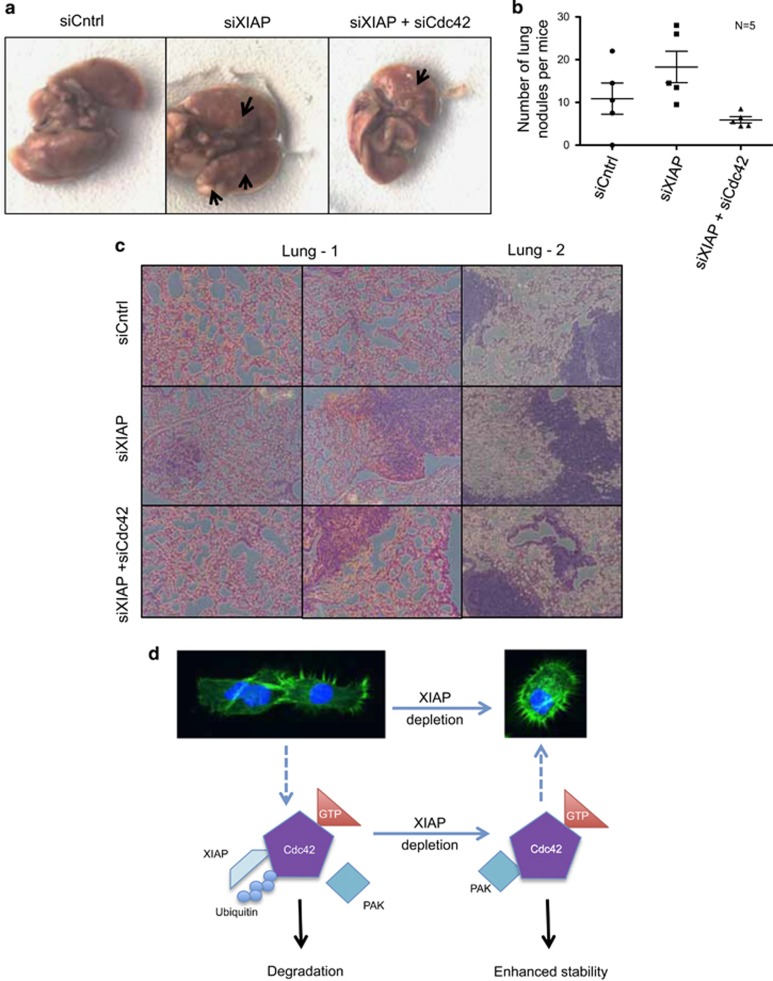
XIAP depletion in tumor cells promotes lung colonization of mice in a Cdc42-dependent manner (**a**) NOD-SCID mice were injected through the tail vein with 1 million tumor cells treated with different siRNAs as indicated. Mice were killed after 4–5 weeks of injection and the lungs were isolated. Shown are pictures of the whole lung (**b**) Metastatic nodules were counted manually on each lung by three independent pairs of eyes, with five mice lungs counted per condition (**c**) Lungs were fixed and H&E-stained and representative images of the lungs are shown. (**d**) Proposed model for the interaction between XIAP and Cdc42

Previous studies have shown that Cdc42 has a crucial role in the metastases of tumor cells.^19^ To evaluate the pathophysiological significance of XIAP-Cdc42 interaction *in vivo*, we employed an experimental metastases model employing NOD/SCID mice. Control or XIAP-depleted HeLa cells were injected into the tail vein of mice, and we detected that there was an increase in the number of nodules in the surface of the lung at 4–5 weeks post injection in mice injected with XIAP-depleted cells (Figure 6a). Co-depletion of Cdc42 strongly reduced the number of lung nodules, confirming that loss of XIAP led to an increase in the number of lung nodules in a Cdc42-dependent manner (Figure 6b). Hemotoxylin and Eosin staining confirmed the presence of nodules in the lungs of the mice (Figure 6c). These results reveal the first E3 ubiquitin ligase of Cdc42 and shed further insights into the molecular mechanism behind XIAP-dependent regulation of actin-rich protrusions and tumor cell invasion.

## Discussion

Rho GTPases are crucial regulators of cell adhesion and migration and the spatiotemporal dynamics of activation has been intensively studied. GEFs and GAPs regulate the activation cycle of Rho GTPases and ubiquitin-dependent inactivation has recently emerged as a ‘non-canonical’ means of Rho GTPase inactivation. RhoA has been shown to be ubiquitinated by Smurf1 and FBXL19 while HACE1, FBXL19 and XIAP/cIAP1 were shown to be the E3 ubiquitin ligases of Rac1 targeting these Rho GTPases for proteasomal degradation.^4, 6, 20, 21, 22^ Rac1 is also SUMOylated by PIAS and this is an essential event for optimal cell migration in response to HGF.^23^ Cdc42 was discovered nearly 25 years ago and its role in regulation of actin cytoskeleton, polarity, cell adhesion and migration is well documented. Cdc42 has been best characterized for its ability to stimulate filopodia formation at the leading edge of the migrating cells. Like other Rho GTPases, binding to RhoGDIs also regulates Cdc42 activity and protein stability. As the activated form of Cdc42 is targeted for ubiquitination and proteasomal degradation, it is possible that interaction with XIAP and ubiquitination is also regulated by interaction with RhoGDI. Further, the RING domain of XIAP has also been shown to interact with RhoGDI.^24^ As XIAP conjugates ubiquitin to the C-terminus, one could envisage that ubiquitinated species of Cdc42 may not interact with RhoGDI. Depletion of XIAP also enhanced the active GTP-bound form of Cdc42 with a concomitant increase in actin-rich filopodial protrusions in normal and tumor cells. As XIAP binds to the activated Cdc42 and promotes ubiquitination, loss of XIAP led to the accumulation of activated Cdc42 in cells. These observations further support the model that XIAP-mediated ubiquitination could serve as a non-canonical way of Cdc42 inactivation. Further studies are clearly warranted to decipher the spatiotemporal dynamics of XIAP-Cdc42 interaction. We observe that XIAP directly interacts with Cdc42 and co-precipitated with the activated form of Cdc42. It would be interesting to test if any other effectors of Cdc42 trigger the RING activity of XIAP.

XIAP is a legitimate caspase inhibitor and thus efforts have been made to target XIAP-caspase inhibition to promote tumor cell apoptosis. Several drugs have been developed to achieve these goals and some of them have reached the clinics. Apart from XIAP, cIAP1 has also been shown to regulate Rho GTPases. cIAP1 can function as an E3 ubiquitin ligase of Rac1 and control Cdc42 activity in response to TNF alpha.^4, 25^ However, we found that loss of cIAP1 did not lead to an increase in the levels of Cdc42. Consistently, we failed to detect any potential ubiquitination of Cdc42 by cIAP1 (Figure 4b). Currently it is unclear about the kind of ubiquitin chains synthesized by XIAP on Cdc42. However, our experiments with TUBEs reveal that polyubiquitination of Cdc42 is dependent on the RING activity of XIAP *in vivo* at endogenous levels. In conclusion, our studies revealed the other side of XIAP in the control of Rho GTPases (especially Rac1 and Cdc42), the prime drivers of tumor cell migration and invasion. Regulation of Cdc42 via GEFs and GAPs is well understood; our study identifies XIAP as the first E3 ubiquitin ligase of Cdc42 and adds another dimension to the inactivation of this Rho GTPase and ubiquitin-dependent regulation of actin cytoskeleton.

## Materials and methods

### Transfection of siRNAs and plasmids

To silence XIAP, cIAP1 and Cdc42 expression by RNA interference, ~75 000 cells/well were seeded in a 12-well plate at least 20 h prior to transfection. siRNAs directed against various genes and scrambled control siRNA as negative control were transfected using Hiperfect (Qiagen, Hilden, Germany) or Saint Red (Synvolux, Leiden, Netherlands) or Lipofectamine RNAimax (Invitrogen, Schwerte, Germany) transfection kits. The transfection was performed according to manufacturer’s instructions. At 24 h post transfection, cells were trypsinized and one half of the cells were seeded out on glass coverslips in a 12-well plate for immunofluorescence analysis if needed, while the other half was seeded for western blot analysis. The cells were normally lysed 48 h post transfection. Unless otherwise mentioned, siRNAs have been transfected at 60 nM. The following siRNAs were employed in this study: XIAP3’-UTR siRNA: Target Sequence: CTGACTGATCTAATTGTATTA (Qiagen), XIAP siRNA-2: Target Sequence: GAAGGAGAUACCGUGCGGUGCUUUA (Invitrogen), XIAP siRNA-3: Target Sequence: AAGTGCTTTCACTGTGGAGGA (Qiagen), Control siRNA: Target Sequence: AATTCTCCGAACGTGTCACGT(Qiagen), Cdc42 siRNA-1: Target Sequence: CATCAGATTTGAAATATTTAA (Qiagen), Cdc42 siRNA-2: Target Sequence: GGCGATGGTGCTGTTGGTAAA (Qiagen).

In the case of overexpression or complementation, plasmids at a stock concentration of 1*μ*g/ml were taken and transfected into cells using PEI transfection reagent. A day after transfection was sufficient to observe complemented proteins in the tumor cells. In the case of Cycloheximide chases post overexpression, the chase experiments were started 40 h post transfection so as to lyse cells 48 h post transfection. For stable shRNA-mediated knockdown Lentiviral particles (mission shRNA) carrying control (Sigma, Taufkirchen, Germany) and Cdc42 shRNA, Sequence: CCGGCCTGATATCCTACACAACAAACTCGAGTTTGTTGTGTAGGATATCAGGTTTTTG, were obtained from Sigma and the stable cell lines were produced following the manufacturer’s instructions. In case of co-knockdown experiments, cells were transfected with siRNAs at a final concentration of 60 nM each.

### Rho GTPase activation assays

XIAP was knocked down in HeLa and HMEC cells and 48 h post transfection, cells were lysed. EGF treatments were performed at the times indicated prior to lysis of cells. The GTP-bound Cdc42 was precipitated using the Cdc42 Active PD kit from Thermo Fisher Scientific, Darmstadt, Germany. This was done according to manufacturer’s instructions.

### Ubiquitylation assays

*In vitro* ubiquitylation of Cdc42 was performed with Cdc42 Wild Type, Cdc42Q61L or Cdc42T17N purified from bacteria. The ubiquitylation reaction was performed in the presence of Ubiquitylation buffer (50 mM Tris-HCl, pH7.5, 100 mM NaCl, 2.5 mM MgCl_2_, 1 mM DTT), 100 nM XIAP (R&D Systems, Minneapolis, MN, USA), 100 nM E1 (Boston Biochem, Cambridge, MA, USA), 150 mM UbcH5a (Boston Biochem), 107 mM His–Ubiquitin (Boston Biochem), 50 mM EDTA, 1 × Mg-ATP (Enzo Life Sciences, Lörrach, Germany), 1U inorganic pyrophosphatase (Fluka, Taufkirchen, Germany) and 50 mM DTT. The reaction was incubated at 37 °C for 30 min. For western blot analyses, the reaction was stopped using Laemmli buffer and Cdc42 was visualized using Cdc42 antibody (BD, Heidelberg, Germany).

### Immunofluorescence and counting of filopodia

Cells were seeded and transfected in 12-well plates. One day post transfection, they were split and re-seeded upon coverslips in a new 12-well plate. The confluency of cells on the cover slip was maintained below 30%. Two days post transfection, cells were taken and washed thrice with PBS before being fixed in 4% paraformaldehyde for 10 min. They were then permeabilised using 0.1% Triton X-100 for 3 min and then blocked for 20 min at RT using 1% BSA in PBS. The actin cytoskeleton was then labeled with 1:50 Alexa Fluor 488 Phalloidin (Invitrogen) in blocking buffer for 20 min in the dark. For staining of Cdc42 protein, cells were incubated in 1:250 dilution of Cdc42 antibody (BD) overnight in 0.5% BSA in PBS. Cells were then incubated with secondary Cy3 antibody in Blocking Buffer for 1 h at RT in the dark. Between each step, the cover slip was always washed thrice with PBS. Finally, the cells were mounted on glass slides using Moviol (Sigma) and examined using a Confocal microscope (Leica (Wetzlar, Germany) TCS SP8). Cells were normally viewed at × 63 magnification and digital zooming was done in case single cells were focused on. Filopodia were then counted manually using the KatiKati software, which has an in-built zoom function to enlarge the images. This was done for all the cell types, tumor and primary cell lines employed in the paper.

### Cell culture

HeLa, BT474 and MDA-MB231 cells were cultured in DMEM medium (Gibco, Schwerte, Germany) supplemented with 10% FCS (Gibco) and 0.2% Penicillin (100 U/ml)/streptomycin (100 *μ*g/ml) (Gibco) at 37 °C in 5% CO_2._ NCI-H226 cells were cultured in RPMI (Gibco) medium supplemented with 10% FCS and antibiotics, while HMEC and HMEC-T cells were cultured in MEGM medium (Lonza, Cologne, Germany) supplemented with growth factors as recommended by the company. For treatment with EGF (Labgen, Germany), a concentration of 25 pg/*μ*l was used. MG132 treatment (Calbiochem, Darmstadt, Germany) was performed 5 h before lysing of cells at a concentration of 10 *μ*M. Cycloheximide chases were performed with InSolution Cycloheximide (Calbiochem) at a final concentration of 100 *μ*g/ml.

### Western blot analysis

Cells were lysed in 5 × Laemmli Buffer and boiled at 100 °C for 5 min before loading them on the polyacrylamide gels. After separation by SDS-PAGE, the proteins were transferred to either nitrocellulose or PVDF membranes (GE Healthcare Life Sciences, Freiburg, Germany). Following the transfer, membranes were then blocked using 5% low fat milk in either phosphate-buffered saline or TBST or or 3%BSA+TBST for 1 h at room temperature. They were subsequently incubated overnight with various primary antibodies diluted in 3% blocking buffer, 3% BSA+TBST or 1% BSA+TBST. Horseradish peroxidase coupled secondary antibodies followed by chemiluminescence (Amersham Biosciences, Millipore, Freiburg, Germany) were used to detect the antigen antibody complexes. Quantification of the obtained western blots was performed by densitometry on ImageJ.

The following antibodies were used in this study: Anti-Cdc42 mouse monoclonal (BD Transduction), Anti-beta Actin (HRP) (Abcam), Anti-beta-actin rabbit polyclonal (Sigma), Anti-XIAP mouse monoclonal (BD Pharmingen), Anti Flag (HRP) (Sigma), Anti-c-Myc mouse monoclonal (Santa Cruz), Anti-GST (Santa Cruz).

### Protein–protein interaction assays

Recombinant XIAP, Cdc42 Wild Type, Cdc42Q61L and Cdc42T17N were expressed as GST fusion proteins in BL-21 *Escherichia coli* strain (Stratagene, Cedar Creek, TX, USA) according to manufacturer’s instructions. The proteins were purified using standard protocols. Proteins were cleaved, if required from their GST tag by using Thrombin (1U) (GE Healthcare). Thrombin cleavage was performed using standard protocols. For the pulldown assays, GST PAK-PBD (Cytoskeleton) was added to the *in vitro* Ubiquitylation reaction of Cdc42 and the mix was incubated at 4 °C for 2 h on the rotator. A total of 10 *μ*l of Glutathione Sepharose Beads (GE Healthcare Life Sciences) which were first washed thrice with Binding Buffer (25 mM Tris-HCl pH7.2, 150 mM NaCl, 0.5% NP-40, 1 mM MgCl_2_, 5% Glycerol) were then added to the reaction mix and was incubated for 1.5 h at 4 °C on the rotator. The beads were then washed thrice with 500 *μ*l of binding buffer and pelleted down at 2500 rpm for 1 min. A total of 50 *μ*l of sample buffer was added before boiling the samples at 100 °C for 5 min. The interaction between the proteins of interest was then observed by SDS-PAGE.

293 T cells were transfected with Cdc42 Wild Type plasmid and cells were harvested 72 h post transfection. RIPA buffer supplemented with phosphatase and protease inhibitors was used to lyse the cells. Lysates were then added on top of various GST tagged XIAP mutants purified in the laboratory and incubated for 2 h at 4 degrees. The beads were subsequently washed and finally lysed in Laemmli buffer. Samples were taken for Western Blot analysis and the GST tagged XIAP mutants were visualized using anti-GST antibody (Santa Cruz), whereas Cdc42 was visualized using anti-Cdc42 antibody (BD).

To check for the nucleotide dependence of binding, GST protein or GST tagged XIAP was first immobilized on Glutathione Sepharose beads and rotated at 4° for 1 h. One microgram of GST tagged XIAP was used for this experiment per sample. The samples were then blocked using 1%BSA in Binding Buffer (25 mM Tris-HCl pH7.2, 150 mM NaCl, 0.5% NP-40, 1 mM MgCl_2_, 5% Glycerol). After 1 h of rotation at 4°, the samples were washed thrice with Binding Buffer and cleaved protein was added on the beads. In all cases, either 1 *μ*g or 2 *μ*g of protein was used. Samples were rotated for 1.5 h at 4 degrees and then washed three times with binding buffer. In this step, 250 mM NaCl is used to minimize background binding. Laemmli buffer was added and samples were heated at 100 °C for 5 min. The interaction between the proteins of interest was then observed by SDS-PAGE.

### HIS-TUBE pulldown

HeLa cells were transfected in a 24-well plate with XIAP siRNA using Lipofectamine RNAiMAX as per manufacturer’s instruction. Cells were transferred to 100mm tissue culture plates 24 h later. 48 h post transfection, cells were treated with MG132 (10 *μ*M) for 6 h. Then cells were lysed in RIPA buffer (250 mM NaCl, 50 mM Tris-HCl, 10% Glycerin, 1% Triton X-100, pH7.5), supplemented with EDTA free Protease Cocktail inhibitor (Roche, 64693159001), and *N*-ethyl maleimide(5 mM). Equal amounts of pre-cleared lysates were incubated with 100 *μ*g/m His6-TUBE1 (LifeSensors, UM201) for 15 min on ice. After collecting total cell lysates, the remaining lysate were incubated in Ni-NTA beads (Qiagen, Ni-NTA Superflow, 30410) and rotated overnight (cold room). Samples were washed three times with RIPA buffer containing 50 mM imidazole and eluted in SDS buffer containing laemmli and analyzed by SDS-PAGE and immunoblotted for Cdc42 using anti-Cdc42 antibody.

### Mass spectrometry

Samples were analyzed using Mass Spectrometric approaches as described in ref. 4

### RT-PCR

Total RNA was extracted from cells using Trizol (Ambion/Thermo Fisher, Schwerte, Germany) or an RNA isolation kit (Thermo Fisher). In brief, cells were resuspended in Trizol, Chloroform was then added to the cell suspension and cells were vortexed for 15 s. They were then spun down at 14 000 rpm for 15 min at 4 °C. The aqueous upper phase was then transferred to sterile tubes containing Isopropanol. After incubating this mix at RT for 10 min, the samples were again spun down at 14 000 rpm for 15 min at 4 °C. They were then washed with 75% Ethanol and spun down at 10 000 rpm for 5 min at 4 °C. The pellet is air dried and resuspended in 50 *μ*l of RNase free dH_2_O. The amount of RNA was subsequently measured via NanoDrop (Thermo Fisher). When using the RNA isolation kit, standard protocol was followed. cDNA was synthesized using the RevertAid RT kit (Thermo Scientific) and manufacturer’s instructions were followed. For PCR amplification, the following primers were used: Cdc42-For-1 – AGTGTTCTGCACTTACACAGAAAG, Cdc42-Rev-1 – CTGCGGCTCTTCTTCGGT, Cdc42-For-2 – AGGCTGTCAAGTATGTGGAGTG, Cdc42-Rev-2 – GGCTCTTCTTCGGTTCTGG, Cdc42-For-3 – CATCGGAATATGTACCGACTGTT, Cdc42-Rev-3 – TGCAGTATCAAAAAGTCCAAGAGTA, GAPDH-For-1 – TGCACCACCAACTGCTTAGC, GAPDH-Rev-1 – GGCATGGACTGTGGTCATGAG, RPS13-For-1 – CGAAAGCATCTTGAGAGGAACA, RPS13-Rev-1- TCGAGCCAAACGGTGAATC.

The qPCR machine was programmed as follows: 1 cycle at 50 °C – 2 min, 50 cycles at 95 °C – 15 min, 95 °C – 15 s, 57 °C – 15 s, 72 °C – 15 s and finally 1 cycle at 95 °C – 1 min.

### Lung colonization experiments

HeLa cells were transfected with the desired siRNAs. Forty-eight hours post transfection, cells were detached from culture plates, and 10^6^ cells were suspended in 100 *μ*l of PBS before injection into the tail veins of NOD-SCID mice. After 5 weeks, lungs were fixed in 4% PFA and analyzed for the presence of surface metastatic foci. Three independent pairs of eyes counted the foci to ensure impartiality of counts. Lungs were then hematoxylin and eosin stained and scanned.

### Statistical analysis

Where applicable, data is expressed, as mean±S.E.M. Statistical significance was determined using an unpaired Student’s *t*-test. In the event where one value was unchanged (as in Figure 1g), a paired *t-*test was used to determine significance.
