# Supplementary material for: Ubiquitin-dependent regulation of Cdc42 by XIAP
Source: Cell Death Dis. 2017 Jun 29;8(6):e2900–. doi: 10.1038/cddis.2017.305 (PMC5520948; doi:10.1038/cddis.2017.305)
Supplement: Supplementary Figures Legends [file cddis2017305x1.docx]

**Supplementary Figure 1:**

(A) Immunofluorescence images of HMEC-T and NCI-H226 cells depleted of XIAP and Phalloidin stained. (B) Cdc42 was stably knocked down in HeLa cells and a transient depletion of XIAP was performed to check for the dependence of filopodia formation on Cdc42. Cells were lysed 48 hours post transfection and fixed and stained as described in Materials & Methods (C) Quantification of Figure (B), 7-8 cells were counted per condition, p<0.001 (D) HMEC-T cells were depleted of XIAP as well as Cdc42 siRNA to look for the dependency of filopodia formation on the two proteins. Cells were fixed and stained (as described in Materials and Methods) with phalloidin.

**Supplementary Figure 2:**

(A) 293T cells were overexpressed with Cdc42 WT plasmid. Cells were lysed 48 hours post overexpression and the lysates were added on different mutants of XIAP (GST tagged). A GST Pulldown was then performed to check for interaction between Cdc42 and the XIAP mutants (B) In-vitro ubiquitination of Cdc42 mutants by XIAP. Purified recombinant Cdc42 Wild Type, Q61L and T17N were subjected to in-vitro ubiquitination by recombinant human XIAP (protocol described in Materials and Methods) (C) Different myc tagged Cdc42 mutants were overexpressed in HeLa cells and a cycloheximide chase was performed to check for half life of protein expression. (D) Myc tagged Cdc42Q61L and Cdc42Q61LK166A mutants were overexpressed in HeLa cells and a cycloheximide chase were performed to check for the half-life of Cdc42 protein.
