# Supplementary figures and images for: Ubiquitin-dependent regulation of Cdc42 by XIAP
Source: Cell Death Dis. 2017 Jun 29;8(6):e2900–. doi: 10.1038/cddis.2017.305 (PMC5520948; doi:10.1038/cddis.2017.305)

# Supplementary Fig.1

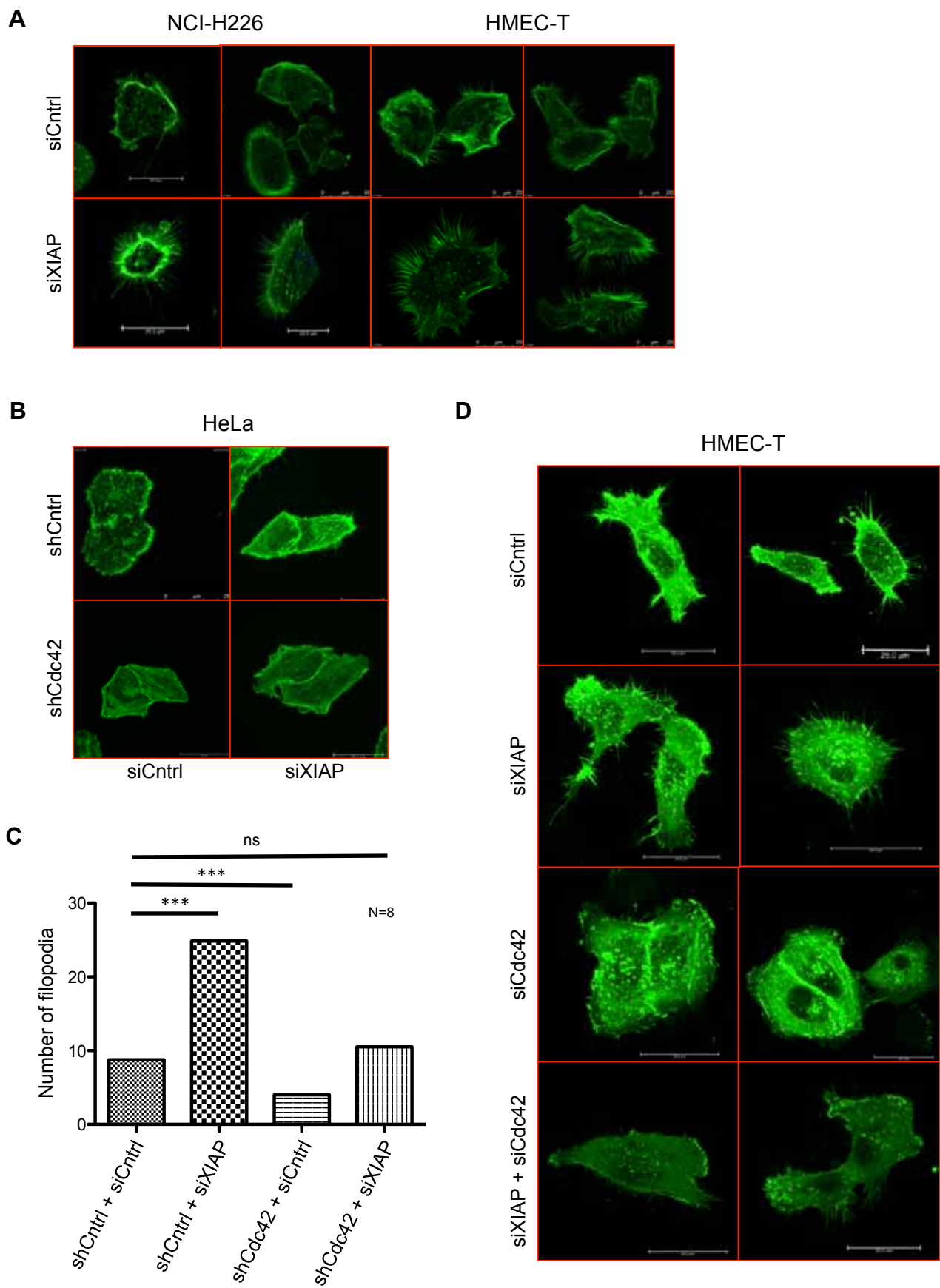

Supplementary Fig.2

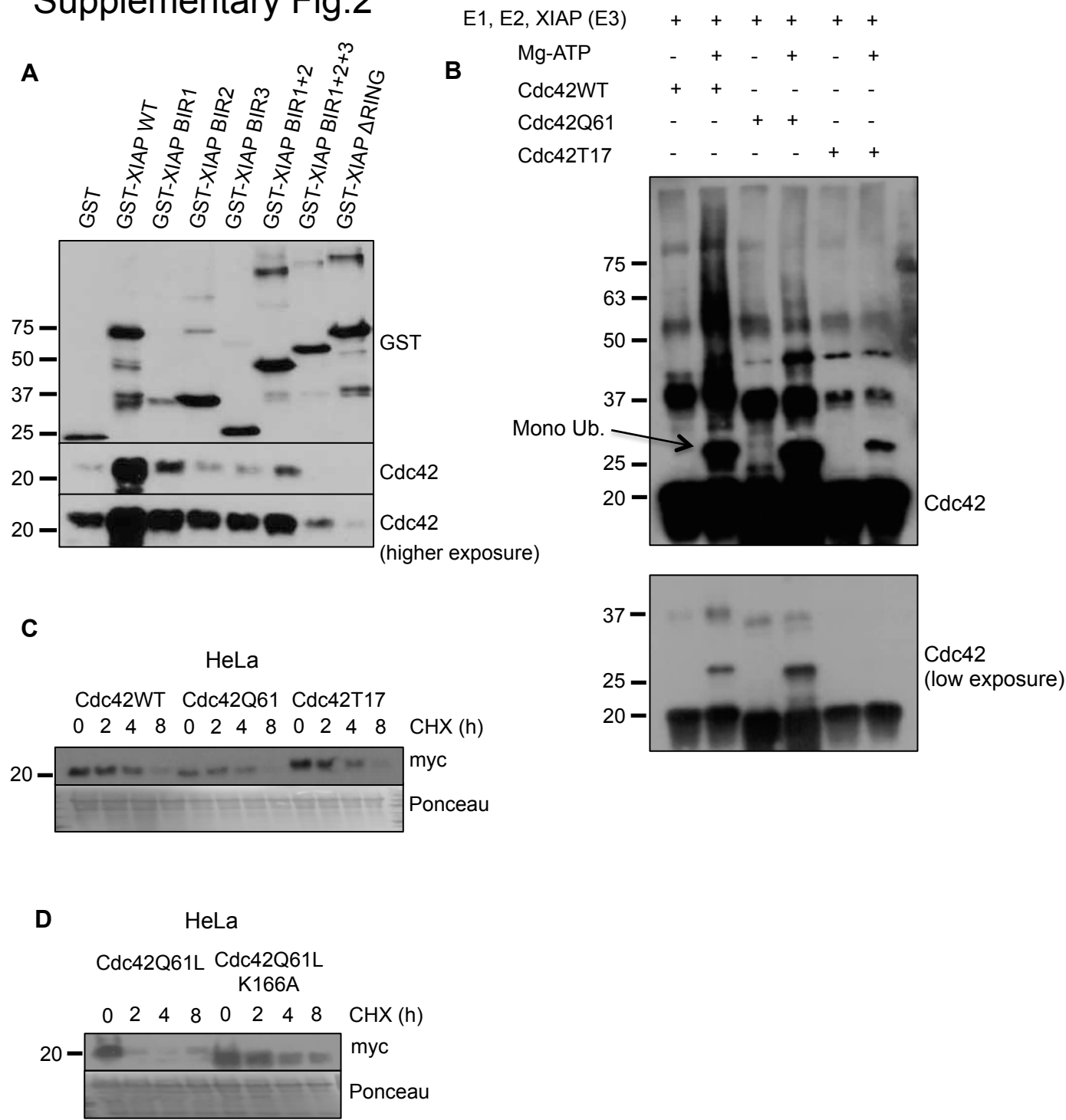

Supplement: Supplementary Figures [file cddis2017305x2.pdf]
